# Strategic Tools in Regenerative and Translational Dentistry

**DOI:** 10.3390/ijms20081879

**Published:** 2019-04-16

**Authors:** Marco Tatullo, Bruna Codispoti, Francesco Paduano, Manuel Nuzzolese, Irina Makeeva

**Affiliations:** 1 Department of Regenerative Medicine, Tecnologica Research Institute, 88900 Crotone, Italy; bruna.codispoti@tecnologicasrl.com (B.C.); francesco.paduano@tecnologicasrl.com (F.P.); 2 Department of Experimental Medicine, Marrelli Hospital, 88900 Crotone, Italy; 3 Department of Therapeutic Dentistry, I.M. Sechenov First Moscow State Medical University, 119435 Moscow, Russia; irina.makeeva@libero.it; 4 Department of NHS Foundation Trust, University Hospitals Birmingham – NHS Foundation Trust, Birmingham B152GW, UK; manuelnuzzolese@libero.it

**Keywords:** oral-derived stem cells, regenerative medicine, dental pulp stem cells, waste medicine, tissue engineering

## Abstract

Human oral-derived stem cells can be easily obtained from several oral tissues, such as dental pulp, periodontal ligament, from gingiva, or periapical cysts. Due to their differentiation potential, oral-derived mesenchymal stem cells are promising for tissue engineering and regenerative medicine. The regenerative ability showed by some oral tissues strongly depends on their sleeping adult stem cell populations that are able to repair small defects and to manage local inflammation. To date, researchers are working on effective and efficient methods to ensure safe and predictable protocols to translate stem cell research into human models. In the last decades, the challenge has been to finally use oral-derived stem cells together with biomaterials or scaffold-free techniques, to obtain strategic tools for regenerative and translational dentistry. This paper aims to give a clear point of view on state of the art developments, with some exciting insights into future strategies.

## 1. Introduction 

Cryopreserving children’s teeth or adult wisdom teeth is gaining worldwide interest for several reasons. Several tissues-banks have been widely disseminated in many countries, with a growing interest, particularly in the most developed countries. Moreover, scientific research has gained attention from patients, often requiring information about the regenerative procedures carried out with dental pulp mesenchymal stem cells.

Despite the fact that the use of mesenchymal stem cells is well known and widely debated, scientific literature still reports several ethical issues related to tissue engineering. Authors pay attention to the use of specific cell types, such as fetal cells, but concerns on xenogeneic cells have been raised too [1]. 

Authors’ believe that storing and banking dental tissues holds promise for the future cell-based dental therapies: such innovative treatments may develop interesting applications in both restorative dentistry [2] and oral oncology [3].

In this context, the main limitations may be related to the in vitro procedures, recognized as “cell manipulation”, that are subjected to regulatory and technical issues. 

Although isolation techniques have been highly improved and simplified, concern remains about the use of cell biology protocols by dental practitioners [4]. More reliable knowledge should also be disseminated regarding the potential effects of such cell therapies: social media has a significant impact on mass-knowledge, in fact, everyone manages one or more profiles on social networks, and it is easy to find websites that would promise almost “miraculous” applications of stem cells for medical use. On the other hand, many recent serious and reliable research studies have strongly demonstrated the therapeutic potentials of dental pulp stem cells on previously untreatable pathologies: it is the case for corneal degeneration or neurological diseases. Interestingly, investigations into newly discovered populations of immature progenitor/stem cells, such as human periapical cysts-MSCs (hPCy-MSCs), have clearly indicated their potential in the treatment of Parkinson’s disease. The use of dental-derived stem cells has been widely reported in the last years, attracting interest from both clinicians and the general population, and their use will be certainly improved by the next generations of dentists who will be more skilled in cell biology and tissue engineering, thus reinforcing the strategic sector of regenerative dentistry [5]. 

**Figure 1:**
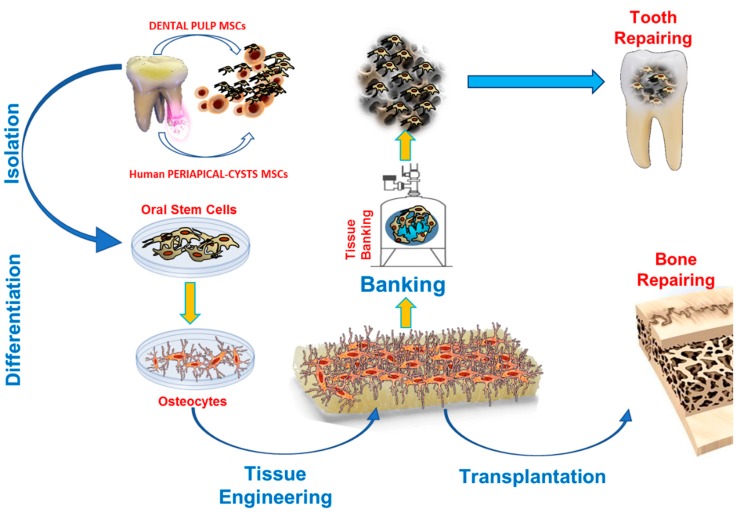
Workflow of the main tasks in the ODSCs processing for regenerative medicine purposes.

Here we propose a general summary of the main research strategies applicable to dentistry: this paper aims to give a clear point of view on state of the art developments, with some exciting insights to those future scientific developments with the highest potential to effectively improve clinical treatments and surgical procedures in the forthcoming years (Figure 1).

## 2. Stem Cells from Oral Tissues

Human oral-derived stem cells (ODSCs) can be obtained from several areas of the mouth, including the inner layer of dental pulp and periodontal tissues. Due to their plasticity and to their great proliferative potential, these mesenchymal stem cells (MSCs) are a promising tool for tissue engineering and regenerative medicine applications [6].

Despite mesenchymal stem cells resident in bone marrow being among the most studied and clinically relevant adult stem cells [7,8,9], in the last years, ODSCs have been reported to show plasticity and proliferation abilities ranging from 30 to 50% higher, with better immunoregulatory behavior compared to bone marrow stem cells (BMSCs) [10].

Isolation of MSCs from human bone marrow (hBM) requires quite invasive procedures. Donor must undergo aspiration of hBM from the iliac crests—hBM-derived stem cells derived from this site are usually not particularly abundant because of their low distribution in hBM [11]. These concerns encouraged scientific research to find alternative sources of MSCs, more available and consistent, to decrease the impact of surgical harvesting of MSCs on patients’ tissues, and to obtain an increased number of stem cells from these newly available sources. 

In recent years, several cell populations (DPSCs, SHED, DFPCs, PDLSCs, SCAPs, hPCy-MSCs) have been reported to show the typical features characterizing MSCs. In the mouth, researchers have found many sites hosting MSCs, thus attracting real interest towards dental tissues and surrounding soft and hard supporting structures [5].

Teeth extraction for orthodontic and infective reasons [12,13], or due to syndromic anatomical abnormalities [14,15], is currently a safe and evidence-based practice in dentistry. The oral cavity is easily accessible compared to other sources of stem cells. Moreover, tissues collected from oral surgery can be directly processed after surgical procedures, to obtain stem cells for regenerative purposes. 

Dental pulp stem cells (DPSCs) are among the most investigated ODSCs. Such cells are able to naturally differentiate towards odontoblasts during such reparative processes involving dentin and other hard structures of teeth [16,17,18,19]. Previously reported in vitro procedures demonstrated that DPSCs always showed the typical phenotypic pattern of MSCs. In fact, such cells showed high expression of CD90, CD73, CD105, CD29, CD13, and CD44 surface antigens, and lacked the expression of hematopoietic markers, including CD34, CD45, and HLA-DR [17]. 

Numerous studies have described the potential of DPSCs to differentiate not only towards the three main stromal lineages (bone, fat, and cartilage) but also towards hepatocytes, myocytes, and neurons [20]. In the light of their self-renewal ability and their vast plasticity, these cells can cover a broad spectrum of clinical applications both in medicine and dentistry. 

More than 20 years of extensive investigations on novel sources of MSCs in oral tissues led to the discovery of a significant number of new populations of adult stem and progenitor cells in both soft and hard oral tissues.

Miura et al. isolated for the first time multipotent, clonogenic, and highly proliferating progenitors from the dental pulp of human exfoliated deciduous teeth (SHED). These cells were capable of repairing dentin and of regenerating damaged bone, in animal models. Furthermore, SHEDs have been shown to differentiate into various cell phenotypes, such as odontoblasts, adipocytes, and neural cells. These cells have been reported to express neural markers in experimental conditions. In fact, SHEDs were successfully differentiated into neuron-like cells and transplanted into the brains of mice, showing a good proliferation rate in such heterotopic environment [21]. 

The dental follicle is a connective sac that surrounds the developing tooth: it contains a population of MSCs, called dental follicle progenitor cells (DFPCs). These cells can start neural differentiation and regenerate both periodontal structures and bone tissue [22]. 

The periodontal ligament is the part of connective tissue that binds together dental cementum and the alveolar bone. This structure houses a cell population that expresses mesenchymal stem-like markers, these cells are termed “stem cells of the periodontal ligament” (PDLSCs). PDLSCs can differentiate into adipocytes, collagen-forming cells, and cementoblasts in vitro. They can also regenerate dental cementum and periodontal ligament in several animal models [23]. Human parotid glands contain cells capable of forming colonies under culture conditions; these cells express both mesenchymal and epithelial markers [24]. Another MSCs population has been found in the jaw periosteum, which can differentiate into osteoblasts and chondrocytes [25]. Gingival tissues also hold clonogenic progenitor cells. Such cells have clearly demonstrated better regenerative potential than the most appreciated BMSCs [26]. Apical papilla is a specific part of the gingiva, located between two adjacent teeth. This tissue is highly vascularized, and colonized by one particular stem cell population, stem cells from apical papilla (SCAPs) that are able to differentiate not only into odontogenic and osteogenic progenitors but also into adipose and neural cells [27]. 

In the last years, ethical and regulatory issues have been highly considered in stem cell research, driving researchers to find new anatomical sources to collect MSCs reducing donor site morbidity. There are several examples of novel and alternative sources of stem cells. These strategic reservoirs are even more required by surgeons in regenerative/reparative procedures, given that the well-known MSCs sources, such as bone marrow and adipose tissue, are often difficult to obtain. Nevertheless, many researchers have recently reported surprising results using biological tissues obtained from medical wastes [28]. Worldwide, authors have described stem cells found in the most heterogeneous tissues. Nevertheless, the scientific soundness of such cells has been widely demonstrated, thus validating the novel and smart concept called “waste medicine”, based on the virtuous reusing of discarded biological tissues [29]. 

Recently, Marrelli et al. isolated and characterized mesenchymal stem from the human periapical dental cysts (hPCy-MSCs). This newly discovered cell population exhibited exceptional commitment towards osteogenic and neurogenic lineage [30], creating high expectations related to hPCy-MSCs in many clinical applications [5].

Collected hPCy-MSCs have a strong self-renewal ability, a multi-lineage differentiation potential, and a high proliferative capability, even after 20 passages in the culture medium. Furthermore, these cells share the same immunophenotype as MSCs, together with the commitment towards adipogenic, osteogenic, and chondrogenic lineages. Interestingly, hPCy-MSCs express, at basal levels, some neuronal- and astrocyte-specific proteins, including β-III tubulin and GFAP [31]. 

The use of biological waste in tissue engineering can be considered an atypical tool for autologous medical therapies. Despite its recent use, some applications have been already described in contemporary scientific literature [28]. In this context, discharged periapical cysts derived from bacteria-sustained inflammations could be soon considered an unbelievably valuable resource for regenerative applications, especially in dental surgery, thanks to the abundant presence of highly immature progenitor cells in their inner layer [32].

## 3. In vitro Manipulation of ODSCs: the Good the Bad and the Ugly 

Research into the regenerative properties of ODSCs has recently tried to better understand if dental tissues and systemic organs could be repaired by such dental-derived progenitor cells. Some researchers reported their results highlighting that multipotent cells from dental pulp, obtained from human third molars, exhibited clear commitment towards cell-based therapy for liver diseases [33]. During the last ten years, several research groups have been investigating innovative and safe protocols involving MSCs from the oral cavity for use in tissue engineering and regenerative medicine (TERM) applications. Interesting research has demonstrated that human dental follicle cells derived from wisdom teeth naïvely express the early neural cell marker beta-III-tubulin, and such cells were also able to form beta-III-tubulin-positive neurosphere-like cell clusters on low-attachment cell culture dishes. The isolated neurogenic-committed stem cells expressed Nurr1, NF-M, and Nestin genes, and such cells have been positively used to treat middle cerebral artery occlusions in murine models, thus assessing their potential utility in stroke therapy [34].

The regenerative behavior of many dental tissues depends on their adult stem cell populations, which possess the ability to differentiate into specialized cells [35]. Currently, it is well known that MSCs from wisdom teeth are easily collectible and have been tested as promising candidates for cellular therapy of dental and periodontal diseases. Previous research has reported that MSCs from wisdom teeth can produce mineralized tissues and complex structures similar to dentin, dental pulp, and periodontal ligament in xenograft models [36].

However, the great problem is to obtain a therapeutically significant cell number from one tooth. 

As previously described, teeth host different types of MSC populations. In fact, depending on the harvest site we may isolate MSCs from dental pulp (DPSCs), from periodontal ligament (PDLSCs), from apical papilla (SCAPs), etc. Thus, although the tooth takes up little space, it holds a set of sites where to isolate abundant cells for therapeutic procedures, with the advantage that their harvesting and manipulation can be linked to routine tooth extraction [36].

In this context, the broad heterogeneity of dental-derived MSCs may represent a bias because of their different behavior in proliferating and differentiating. Thus, each MSC isolated should undergo multiple in vitro culturing passages, till a proper amount of cells is reached.

A criticism may be related to the consequences of MSCs being propagated in vitro for long periods. However, recent studies specifically focused on dental stem cells have demonstrated their excellent stability and plasticity, without compromising their normal karyotype, even after 60 population doublings. It has also been demonstrated that the expansion period should not be longer than 20 to 40 population doublings [37].

To date, no isolation technique is preferable, however, researchers have worked on increasingly efficient methods ensuring the maintenance of clinically safe MSCs in sufficient numbers for various regenerative protocols in animal models. Currently, enzymatic digestion of tissues provides high proliferative capacity, good karyotype stability, and a proper in vitro expansion able to obtain a sufficient number of cells for biomedical use [38]. Nevertheless, some isolation protocols are based only on the mechanical mincing of surgical tissues, followed by plating of the obtained small pieces in specific culture media. This mild method could be considered slower in releasing adherent cells on the plate surface, respect to enzymatic digestion [17].

Wisdom teeth represent the best source for isolation of dental pulp cells, but it’s important to emphasize that not all people have wisdom teeth. Therefore, such teeth have limited use for autologous cell therapies. In this light, scientific literature reports several alternatives to wisdom teeth for regenerative applications. Despite each dental derived MSC being autonomously isolated and described, it’s commonly accepted that human dental MSCs from follicle, pulp, and papilla tissue of a single donor show similar morphology, proliferation rate, expression of MSC-specific, and pluripotency markers, and comparable in vitro differentiation ability into the three main mesenchymal lineages [39]. 

Human deciduous teeth are also attractive because of their abundant presence during childhood. Periodontal ligament stem cells from human deciduous teeth (DePDLSCs) combined with human dentin blocks were able to generate new cementum/periodontal ligament-like tissues [40].

Moreover, experimental studies suggested that stem cells from human exfoliated deciduous teeth can differentiate into both odontoblast-like and endothelial-like cells in vivo, thus representing a viable and promising source of stem cells for in vivo dental pulp tissue engineering [41].

### 3.1. ODSCs Applications: from Bench to Dental-Chair Side 

In the last years, studies about teeth-derived stem cells have confirmed their enormous potential in TERM applications. Their use has been reported in several exciting techniques, with substantial impact on general health, demonstrated by research describing how stem cells from wisdom teeth have been transformed into functional corneal cells [42]. In wisdom teeth, researchers have found mesenchymal stem cells easily harvestable and with an enormous range of potential therapeutic applications, such as in the treatment of spinal cord injuries, brain damage, myocardial infarction, wound healing, bone replacement, etc., thus, the scientific community has begun considering them as “a kind of magic” cells [41].

To fully understand the real potential of MSCs in translational therapies, some critical steps should be made. The management of such resource is hardly subjected to issues like those related to manufacturing under GMP conditions, associated with the usefulness of MSCs subjected to banking procedures. It is also important to consider that the regulatory challenges remain discouraging [43]. Stem cell banking of cryopreserved cells may allow readily available transplantable cells with both proliferative/reparative properties and anti-inflammatory/immuno-modulatory behavior. These peculiar characteristics may be urgently requested since from the very early childhood. 

The transplantation of human cells and tissues has become a global challenge for medical and biological purposes. Currently, medical and biological protocols involve numerous ethical and policy issues relating to informed consent, quality, safety of procurement, processing of the biological tissues, and the distribution and international circulation of human cells and tissues. Both ethical and legal recommendations, due to the critical nature of bioethical issues, are mostly variable, often on local and national levels. To create order in the stem cells banking regulatory system, international bodies such as the National Academy of Sciences have published guidelines to stimulate the adherence of multiple global biobanks under minimum universal principles, regarding all the main debated aspects [44].

Human post-natal stem cells can be recovered both from freshly extracted teeth and from cryopreserved dental tissues, such as dental pulp or human periodontal ligament, providing a practical clinical utility for those frozen tissues stored in biobanks for stem cell isolation. Of course, the experienced in vitro manipulation of cryopreserved tissues is crucial for the future clinical use of stem cells, as well as proper protocols for tissue harvesting [45] and decontamination from intraoral bacteria [12,46]. 

A recent research field has developed, aimed at investigating the ability of well-known transcriptional factors in reprogramming human somatic cells obtained from dental pulp to induced pluripotent stem (iPS) cells. In an experimental study, induced pluripotent stem (iPS) cells from human dental pulp of adult and from deciduous teeth were reported to differentiate into neural crest cells, creating a novel source of stem cells for bone and cartilage tissue engineering [47]. Wisdom teeth are easily accessible with minimally invasive procedures, and dental pulp cells are an optimal source of iPS cells, creating optimal conditions to increase the advantages of collecting wisdom teeth as a source of iPS cells for cell banking and use in regenerative medicine.

Banking of human dental pulp from healthy extracted wisdom teeth has been considered for the extreme richness in MSCs or cells useful in iPS technology. However, it’s also interesting to think that dental pulp can be successfully decellularized, and the resulting scaffold behaves as excellent support for the proliferation and differentiation of oral MSCs derived from surrounding tissues after scaffold transplantation [48]. In current scientific literature, the future application of this innovative autograft is mainly addressed to regenerative procedures of dental pulp, thus creating the biological conditions to promote the survival and function of those compromised teeth with otherwise poor prognosis [49].

### 3.2. Scaffolds-Supported Tissue Engineering Protocols

With the spread of cell-based technologies, many of the current tissue engineering methods have taken advantage of the application of scaffolds made of both polymeric and natural biomimetic materials [50].

The supporting role of these biocompatible structures is to provide an adequate three-dimensional environment for cells seeded onto their surfaces, to promote cell proliferation and differentiation into lineages. The physical and mechanical features of biomedical scaffolds may influence the behavior and the differentiation of the hosted stem cells. Mechanical stimuli, including co-axial mechanical stretch, pulsating fluid flow, cyclic tensile strain, low-intensity pulsed ultrasound and topographies of micro- and nano-scale surfaces, combined with external biochemical stimuli, are actively involved in the differentiation fate of DPSCs [51]. Pulp from wisdom teeth has been reported to be an interesting source of MSCs. However, the protocols of tissue engineering need to combine cells and biomaterials to ensure proper tissue repairing/regeneration. 

Several scaffolds have been reported in recent scientific literature; however, only in the last years have the new biomaterials allowed obtaining a useful construct, usable in dental and maxillofacial defects [16]. 

In a recent study, authors reported a composite biomaterial made from a high percentage of propolis extract combined with shell clam: this biomaterial was tested for its antiseptic and osteoinductive skills, to use it in dentistry and maxillofacial surgery. This new biomaterial was able to host human stem cells, ensuring their survival and a good proliferation rate. This scaffold was also osteogenic, thus improving the bone reparation in critical-sized defects [52].

Scaffolds and stem cells have been also applied for the treatment of severe chronic oral inflammations, such as the periodontitis, that may affect the integrity of both hard and soft oral tissues [53]. 

Some approaches exploited the use of biocompatible nanofibers and injectable scaffolds, combined or not with stem cells and/or growth factors, for the intra-pulpal drug delivery for the creation of an environment free from any infective agents, and thus highly favorable for tissue regeneration [54]. 

Furthermore, there are still different issues relating to the long-term safety and effectiveness of these materials. Synthetic polymers are difficult to be controlled in terms of in situ degradation and retention into a surgical site; on the other hand, biological materials could be subjected to bacterial and viral infections; moreover, these bio-scaffolds may potentially trigger immunologic reactions [55]. 

To minimize unknown risks, several research groups have proposed alternative approaches, based on the application of different methodologies that exclude the use of biomaterials. A number of these novel approaches have been able to stimulate the proliferation and/or the differentiation of MSCs.

Although the classic concept of the scaffold is still quite interesting to further develop, some research groups are exploiting the scaffold-free approach, which seems to have promising applications in translational medicine [56]. 

Novel “scaffold-free” technologies are based on the formation of tridimensional cell-to-cell aggregates, in the absence of any other external support.

Scaffold-free engineered cell-made constructs could benefit from the application of external forces to the biological system, such as a rotational force to suspended cells, or cells cultured in non-adherent culturing conditions, creating several cell aggregates. 

Furthermore, the cell-sheet technologies are based on the formation of an adherent monolayer of cells that allows cells to preserve cell–cell junctions, cell surface antigens, and proteins from the extracellular matrix [57]. Cultured cells proliferate up to an overall confluence, and cell sheets can be collected from the surface of tissue culture plate without any enzymatic treatment. In fact, it is enough to use mild methods, such as temperature-responsive cell culture surface plates.

Currently, the treatment of complex bone defects caused by complete fractures, by oncological pathologies, by anatomical deformities, or by syndromic abnormalities, still represents a clinical issue, and several approaches for promoting the bone regeneration are described in the scientific literature [58]. 

The use of autologous bone grafts is considered one of the safest and predictable approaches, representing the gold standard of treating skeletal defects and of regenerating lost bone. The application of allogenic or xenogenic bones, and synthetic substitutes, represent valuable alternatives to autologous implants. However, novel regenerative techniques are highly investigated to limit the use of biomaterials. Recently, several studies have been carried out on 4-(4-methoxyphenyl)pyrido(40,30:4,5)thieno(2,3-b)pyridine-2-carboxamide (TH), a helioxanthin derivative able to promote osteogenesis in MSCs populations. 

These skills have been reported mainly on animal models, nevertheless, the use of TH on human DPSCs from wisdom teeth by using the cell-sheet technology has also been investigated. The obtained results showed that TH improved the osteogenic differentiation of DPSCs in culture, as well as the in vivo osteogenic commitment of TH-treated DPSCs [59].

Other methods include the use of functional molecules able to stimulate and to address cell behavior. Particular interest is currently focused on the adverse effects induced by the modern people’s lifestyle. Indeed, bad alimentary habits, inadequate physical activity, smoking and general stress due to the complexity of working operations, contribute to immunologic alterations and induce a reduction of the MSCs responses. Thus, MSCs are negatively influenced by general lifestyles in indirect manners, and this may impact the quality of tissues reparative/regenerative response [46]. The use of both synthetic and natural antioxidant molecules has been confirmed to exert antitumoral functions, mainly by their capability to mitigate oxidative stress [60]. However, their positive effects also reach stem cell compartments, incrementing immature cell mobilization, maturation, and growing [61]. Finally, a recent work describes an increased wound healing ability of human PDLSCs treated whit cold atmospheric plasma at different exposition settings. This novel approach represents an effective treatment for oral pathologies, independent from the use of any scaffold or biomolecule [62].

### 3.3. Alternative Approaches in Regenerative/Reparative Dentistry 

In the last decades, collecting and banking human MSCs from healthy tissues, such as dental tissues, have been aimed to use such cells, in combination with biomaterials, for tissue-engineering applications. As previously stated, many oral and dental tissues represent easily accessible source of MSCs. Although these immature cells are commonly related to their higher proliferation and stemness, MSCs are also the principal regulatory cells involved in the acceleration of wound healing, in immunologic tolerance in tissue and organs transplantation, and the regulation of body responses in diabetes and other autoimmune diseases. These effects are mainly mediated by the essential paracrine signaling molecules released by MSCs [63]. These molecular mediators promote immunomodulatory and locally strong therapeutic effects, specifically regulatory effects that may be mediated through the release of extracellular vesicles (EVs) that act as paracrine signalers [64]. 

**Figure 2:**
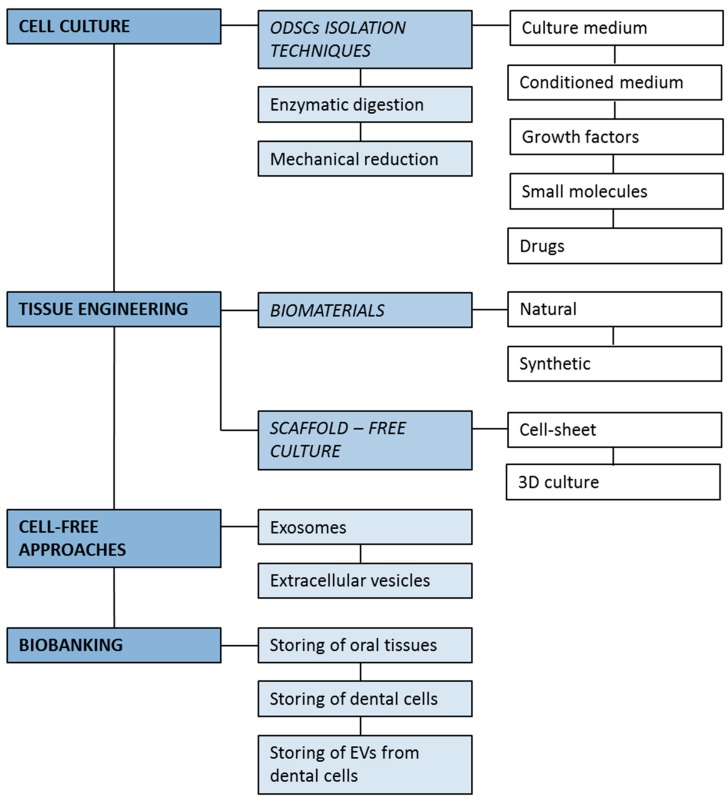
Innovative approaches in regenerative dentistry.

Among EVs, increasing evidence has reported that vesicles with a size of approximately 30–100 nm, called exosomes, play a pivotal role in stem cell therapy, mainly because of their nano-sized phospholipidic bodies, containing cytokines and microRNAs, strategically released in target-tissues [65]. Recent research has reported that the sole presence of exosomes may indicate therapeutic effect in several injury models [66]. This cell-free concept of therapy holds great potential because of its approach safe and with a low-immunogenicity. The future regenerative procedures could be based on exosomes-based cell-free criteria, and dental-derived MSCs may provide the exosomes-rich conditioned medium able to influence and improve the regenerative dental processes. ODSCs have been explored for several applications, and their use has been tested with the most innovative technologies (Figure 2). ODSCs are easily collected with traditional isolation methodologies, and these cells can also be used in combination with a broad spectrum of biomaterials and tridimensional scaffold-free cultures. Moreover, the discovery of extracellular vesicles, such as exosomes, have facilitated storing the precious paracrine molecular pattern of MSCs, offering the possibility of utilizing and applying only the molecular cell effectors of MSCs with a promising cell-free approach. The research on stem cells residing in oral tissues is certainly useful for the numerous translational applications potentially offered. Several protocols for the harvesting and the manipulation of ODSCs are emerging, together with various innovative biological constructs based on the combination of these cells with biomaterials and/or biomolecules. The opportunity to store ODSCs for future autologous or allogeneic use is extremely precious, as they certainly will be a regenerative tool in medical applications.

## 4. Conclusions

Dental derived stem cells have strategically linked clinical dentistry to cell biology. 

Of course, MSCs have been primarily investigated, and sometimes used in innovative treatments, however, the issues related to the site of harvesting have increasingly led research towards more accessible tissues where to find stem cells [67]. In the scientific literature, a growing number of articles are focused on ODSCs application for the regeneration/replacing of soft and hard oral tissues. Currently, a phase-1 clinical trial is ongoing, to clarify the efficiency of autologous PDLSCs to regenerate periodontal tissue in patients affected by periodontitis with deep intraosseous defects (NCT01357785, ClinicalTrials.gov). The last frontier of tissues regeneration has been achieved with the novel concept of the biological waste-based medicine. Here, wasted tissues are a precious resource of biologically active compounds, just like the MSCs. 

Wisdom teeth are commonly considered an annoyance that eventually needs to be removed. Clinically, they have no specific functions, instead, they are often the source of dental pathologies often requiring surgical therapy and complex pharmacological treatments. Even periapical inflamed oral cysts that develop after a chronic inflammation of periodontal tissues in a large number of patients are considered biological waste. However, it has been now discovered that hPCy-MSCs represent a smart source of stem and progenitor cells. This concept can be applied in several other tissues derived from wisdom teeth or human exfoliated deciduous teeth. These ODSCs could be used not only for dental application but also in general regenerative medicine. This approach makes these cells particularly suitable for tissue engineering applications. 

Therefore, the idea of obtaining benefits in terms of MSCs collection thanks to readily available and potentially harmful dental elements represented an excellent motivation that prompted thousands of people to inquire about the possibility of cryo-conserving their oral derived tissues, and those of their children over the last few years. Research in regenerative dentistry is extremely newsworthy and offers the opportunity to effectively revolutionize the old classical dental clinical approaches toward innovative, powerful methodologies.

## References

[1] Volarevic V., Markovic B.S., Gazdic M., Volarevic A., Jovicic N., Arsenijevic N., Armstrong L., Djonov V., Lako M., Stojkovic M. (2018). Ethical and safety issues of stem cell-based therapy. Int. J. Med. Sci..

[2] Lo Giudice G., Lo Giudice R., Matarese G., Isola G., Cicciù M., Terranova A., Palaia G., Romeo U. (2015). Evaluation of magnification systems in restorative dentistry. An in-vitro study. Dent. Cadmos.

[3] Isola G., Cicciu M., Fiorillo L., Matarese G. (2017). Association between odontoma and impacted teeth. J. Craniofac. Surg..

[4] Tatullo M. (2018). About stem cell research in dentistry: Many doubts and too many pitfalls still affect the regenerative dentistry. Int. J. Med. Sci..

[5] Tatullo M., Marrelli M., Paduano F. (2015). The regenerative medicine in oral and maxillofacial surgery: The most important innovations in the clinical application of mesenchymal stem cells. Int. J. Med. Sci..

[6] Morsczeck C., Reichert T.E. (2018). Dental stem cells in tooth regeneration and repair in the future. Expert Opin. Biol. Ther..

[7] Ghiroldi A., Piccoli M., Cirillo F., Monasky M.M., Ciconte G., Pappone C., Anastasia L. (2018). Cell-based therapies for cardiac regeneration: A comprehensive review of past and ongoing strategies. Int. J. Mol. Sci..

[8] Rozier P., Maria A., Goulabchand R., Jorgensen C., Guilpain P., Noel D. (2018). Mesenchymal stem cells in systemic sclerosis: Allogenic or autologous approaches for therapeutic use?. Front. Immunol..

[9] Tatullo M. (2019). Science is not a Social Opinion. Dent. J..

[10] Mantesso A., Sharpe P. (2009). Dental stem cells for tooth regeneration and repair. Expert Opin. Biol. Ther..

[11] Beyer Nardi N., da Silva Meirelles L. (2006). Mesenchymal stem cells: Isolation, in vitro expansion and characterization. Handbook of Experimental Pharmacology.

[12] Marrelli M., Tatullo M., Dipalma G., Inchingolo F. (2012). Oral infection by staphylococcus aureus in patients affected by white sponge nevus: A description of two cases occurred in the same family. Int. J. Med. Sci..

[13] Giudice A., Bennardo F., Barone S., Antonelli A., Figliuzzi M.M., Fortunato L. (2018). Can autofluorescence guide surgeons in the treatment of medication-related osteonecrosis of the jaw? A prospective feasibility study. J. Oral Maxillofac. Surg..

[14] Inchingolo F., Tatullo M., Abenavoli F.M., Marrelli M., Inchingolo A.D., Gentile M., Inchingolo A.M., Dipalma G. (2010). Non-syndromic multiple supernumerary teeth in a family unit with a normal karyotype: Case report. Int. J. Med. Sci..

[15] Figliuzzi M.M., Giudice A., Pileggi S., Pacifico D., Marrelli M., Tatullo M., Fortunato L. (2016). Implant-prosthetic rehabilitation in bilateral agenesis of maxillary lateral incisors with a mini split crest. Case Rep. Dent..

[16] Paduano F., Marrelli M., Alom N., Amer M., White L.J., Shakesheff K.M., Tatullo M. (2017). Decellularized bone extracellular matrix and human dental pulp stem cells as a construct for bone regeneration. J. Biomater. Sci. Polym. Ed..

[17] Tatullo M., Marrelli M., Shakesheff K.M., White L.J. (2015). Dental pulp stem cells: Function, isolation and applications in regenerative medicine. J. Tissue Eng. Regen. Med..

[18] Tatullo M., Falisi G., Amantea M., Rastelli C., Paduano F., Marrelli M. (2015). Dental pulp stem cells and human periapical cyst mesenchymal stem cells in bone tissue regeneration: Comparison of basal and osteogenic differentiated gene expression of a newly discovered mesenchymal stem cell lineage. J. Biol. Regul. Homeost Agents.

[19] Marrelli M., Pujia A., Palmieri F., Gatto R., Falisi G., Gargari M., Caruso S., Apicella D., Rastelli C., Nardi G.M. (2016). Innovative approach for the in vitro research on biomedical scaffolds designed and customized with CAD-CAM technology. Int. J. Immunopathol. Pharmacol..

[20] Iohara K., Zheng L., Ito M., Tomokiyo A., Matsushita K., Nakashima M. (2006). Side population cells isolated from porcine dental pulp tissue with self-renewal and multipotency for dentinogenesis, chondrogenesis, adipogenesis, and neurogenesis. Stem Cells.

[21] Miura M., Gronthos S., Zhao M., Lu B., Fisher L.W., Robey P.G., Shi S. (2003). Shed: Stem cells from human exfoliated deciduous teeth. Proc. Natl. Acad. Sci. USA.

[22] Morsczeck C., Gotz W., Schierholz J., Zeilhofer F., Kuhn U., Mohl C., Sippel C., Hoffmann K.H. (2005). Isolation of precursor cells (PCs) from human dental follicle of wisdom teeth. Matrix Biol..

[23] Seo B.M., Miura M., Gronthos S., Bartold P.M., Batouli S., Brahim J., Young M., Robey P.G., Wang C.Y., Shi S. (2004). Investigation of multipotent postnatal stem cells from human periodontal ligament. Lancet.

[24] Yi T., Lee S., Choi N., Shin H.S., Kim J., Lim J.Y. (2016). Single cell clones purified from human parotid glands display features of multipotent epitheliomesenchymal stem cells. Sci. Rep..

[25] Hutmacher D.W., Sittinger M. (2003). Periosteal cells in bone tissue engineering. Tissue Eng..

[26] Tomar G.B., Srivastava R.K., Gupta N., Barhanpurkar A.P., Pote S.T., Jhaveri H.M., Mishra G.C., Wani M.R. (2010). Human gingiva-derived mesenchymal stem cells are superior to bone marrow-derived mesenchymal stem cells for cell therapy in regenerative medicine. Biochem. Biophys. Res. Commun..

[27] Yang B., Qiu Y., Zhou N., Ouyang H., Ding J., Cheng B., Sun J. (2017). Application of stem cells in oral disease therapy: Progresses and perspectives. Front. Physiol..

[28] Gaggi G., Izzicupo P., Di Credico A., Sancilio S., Di Baldassarre A., Ghinassi B. (2019). Spare parts from discarded materials: Fetal annexes in regenerative medicine. Int. J. Mol. Sci..

[29] Ulrich D., Muralitharan R., Gargett C.E. (2013). Toward the use of endometrial and menstrual blood mesenchymal stem cells for cell-based therapies. Expert Opin. Biol. Ther..

[30] Marrelli M., Paduano F., Tatullo M. (2015). Human periapical cyst-mesenchymal stem cells differentiate into neuronal cells. J. Dent. Res..

[31] Paduano F., Marrelli M., Palmieri F., Tatullo M. (2016). Cd146 expression influences periapical cyst mesenchymal stem cell properties. Stem Cell Rev..

[32] Tatullo M., Codispoti B., Pacifici A., Palmieri F., Marrelli M., Pacifici L., Paduano F. (2017). Potential use of human periapical cyst-mesenchymal stem cells (hPCy-MSCs) as a novel stem cell source for regenerative medicine applications. Front. Cell Dev. Biol..

[33] Ikeda E., Yagi K., Kojima M., Yagyuu T., Ohshima A., Sobajima S., Tadokoro M., Katsube Y., Isoda K., Kondoh M. (2008). Multipotent cells from the human third molar: Feasibility of cell-based therapy for liver disease. Differentiation.

[34] Yang K.L., Chen M.F., Liao C.H., Pang C.Y., Lin P.Y. (2009). A simple and efficient method for generating nurr1-positive neuronal stem cells from human wisdom teeth (tNSC) and the potential of tNSC for stroke therapy. Cytotherapy.

[35] Chen F.M., Sun H.H., Lu H., Yu Q. (2012). Stem cell-delivery therapeutics for periodontal tissue regeneration. Biomaterials.

[36] Racz G.Z., Kadar K., Foldes A., Kallo K., Perczel-Kovach K., Keremi B., Nagy A., Varga G. (2014). Immunomodulatory and potential therapeutic role of mesenchymal stem cells in periodontitis. J. Physiol. Pharmacol..

[37] Suchanek J., Visek B., Soukup T., El-Din Mohamed S.K., Ivancakova R., Mokry J., Aboul-Ezz E.H., Omran A. (2010). Stem cells from human exfoliated deciduous teeth-isolation, long term cultivation and phenotypical analysis. Acta Medica (Hradec Kralove).

[38] Kerkis I., Caplan A.I. (2012). Stem cells in dental pulp of deciduous teeth. Tissue Eng. Part B Rev..

[39] Patil R., Kumar B.M., Lee W.J., Jeon R.H., Jang S.J., Lee Y.M., Park B.W., Byun J.H., Ahn C.S., Kim J.W. (2014). Multilineage potential and proteomic profiling of human dental stem cells derived from a single donor. Exp. Cell Res..

[40] Ji K., Liu Y., Lu W., Yang F., Yu J., Wang X., Ma Q., Yang Z., Wen L., Xuan K. (2013). Periodontal tissue engineering with stem cells from the periodontal ligament of human retained deciduous teeth. J. Periodontal Res..

[41] Cordeiro M.M., Dong Z., Kaneko T., Zhang Z., Miyazawa M., Shi S., Smith A.J., Nor J.E. (2008). Dental pulp tissue engineering with stem cells from exfoliated deciduous teeth. J. Endod..

[42] (2015). Stem cells from wisdom teeth transformed into corneal cells. Dent. Today.

[43] Thirumala S., Goebel W.S., Woods E.J. (2013). Manufacturing and banking of mesenchymal stem cells. Expert Opin. Biol. Ther..

[44] Dricu A. (2018). Recent challenges with stem cell banking. Expert Opin. Biol. Ther..

[45] Marrelli M., Paduano F., Tatullo M. (2013). Cells isolated from human periapical cysts express mesenchymal stem cell-like properties. Int. J. Biol. Sci..

[46] Marrelli M., Gentile S., Palmieri F., Paduano F., Tatullo M. (2014). Correlation between surgeon’s experience, surgery complexity and the alteration of stress related physiological parameters. PLoS ONE.

[47] Kawano E., Toriumi T., Iguchi S., Suzuki D., Sato S., Honda M. (2017). Induction of neural crest cells from human dental pulp-derived induced pluripotent stem cells. Biomed. Res..

[48] Hu L., Gao Z., Xu J., Zhu Z., Fan Z., Zhang C., Wang J., Wang S. (2017). Decellularized swine dental pulp as a bioscaffold for pulp regeneration. BioMed Res. Int..

[49] Tamaoki N., Takahashi K., Tanaka T., Ichisaka T., Aoki H., Takeda-Kawaguchi T., Iida K., Kunisada T., Shibata T., Yamanaka S. (2010). Dental pulp cells for induced pluripotent stem cell banking. J. Dent. Res..

[50] Aulino P., Costa A., Chiaravalloti E., Perniconi B., Adamo S., Coletti D., Marrelli M., Tatullo M., Teodori L. (2015). Muscle extracellular matrix scaffold is a multipotent environment. Int. J. Med. Sci..

[51] Marrelli M., Codispoti B., Shelton R.M., Scheven B.A., Cooper P.R., Tatullo M., Paduano F. (2018). Dental pulp stem cell mechanoresponsiveness: Effects of mechanical stimuli on dental pulp stem cell behavior. Front. Physiol..

[52] Simu M.R., Pall E., Radu T., Miclaus M., Culic B., Mesaros A.S., Muntean A., Filip G.A. (2018). Development of a novel biomaterial with an important osteoinductive capacity for hard tissue engineering. Tissue Cell.

[53] Bottino M.C., Pankajakshan D., Nor J.E. (2017). Advanced scaffolds for dental pulp and periodontal regeneration. Dent. Clin. N. Am..

[54] Pankajakshan D., Albuquerque M.T., Evans J.D., Kamocka M.M., Gregory R.L., Bottino M.C. (2016). Triple antibiotic polymer nanofibers for intracanal drug delivery: Effects on dual species biofilm and cell function. J. Endod..

[55] Daniels A.U., Andriano K.P., Smutz W.P., Chang M.K., Heller J. (1994). Evaluation of absorbable poly(ortho esters) for use in surgical implants. J. Appl. Biomater..

[56] DuRaine G.D., Brown W.E., Hu J.C., Athanasiou K.A. (2015). Emergence of scaffold-free approaches for tissue engineering musculoskeletal cartilages. Ann. Biomed. Eng..

[57] Shimomura K., Ando W., Fujie H., Hart D.A., Yoshikawa H., Nakamura N. (2018). Scaffold-free tissue engineering for injured joint surface restoration. J. Exp. Orthop..

[58] Paduano F., Marrelli M., Amantea M., Rengo C., Rengo S., Goldberg M., Spagnuolo G., Tatullo M. (2017). Adipose tissue as a strategic source of mesenchymal stem cells in bone regeneration: A topical review on the most promising craniomaxillofacial applications. Int. J. Mol. Sci..

[59] Fujii Y., Kawase-Koga Y., Hojo H., Yano F., Sato M., Chung U.I., Ohba S., Chikazu D. (2018). Bone regeneration by human dental pulp stem cells using a helioxanthin derivative and cell-sheet technology. Stem Cell Res. Ther..

[60] Tatullo M., Simone G.M., Tarullo F., Irlandese G., Vito D., Marrelli M., Santacroce L., Cocco T., Ballini A., Scacco S. (2016). Antioxidant and antitumor activity of a bioactive polyphenolic fraction isolated from the brewing process. Sci. Rep..

[61] Shaban S., El-Husseny M.W.A., Abushouk A.I., Salem A.M.A., Mamdouh M., Abdel-Daim M.M. (2017). Effects of antioxidant supplements on the survival and differentiation of stem cells. Oxid. Med. Cell. Longev..

[62] Kleineidam B., Nokhbehsaim M., Deschner J., Wahl G. (2019). Effect of cold plasma on periodontal wound healing-an in vitro study. Clin. Oral Investig..

[63] Wang M., Yuan Q., Xie L. (2018). Mesenchymal stem cell-based immunomodulation: Properties and clinical application. Stem Cells Int..

[64] Rajan T.S., Giacoppo S., Diomede F., Ballerini P., Paolantonio M., Marchisio M., Piattelli A., Bramanti P., Mazzon E., Trubiani O. (2016). The secretome of periodontal ligament stem cells from MS patients protects against EAE. Sci. Rep..

[65] Zomer A., Vendrig T., Hopmans E.S., van Eijndhoven M., Middeldorp J.M., Pegtel D.M. (2010). Exosomes: Fit to deliver small RNA. Commun. Integr. Biol..

[66] Codispoti B., Marrelli M., Paduano F., Tatullo M. (2018). Nanometric bio-banked MSC-derived exosome (nanobiome) as a novel approach to regenerative medicine. J. Clin. Med..

[67] Di Vito A., Giudice A., Chiarella E., Malara N., Bennardo F., Fortunato L. (2019). In vitro long-term expansion and high osteogenic potential of periodontal ligament stem cells: More than a mirage. Cell Transplant..

